# Requirements for MRN endonuclease processing of topoisomerase II-mediated DNA damage in mammalian cells

**DOI:** 10.3389/fmolb.2022.1007064

**Published:** 2022-09-23

**Authors:** Yilun Sun, Eroica Soans, Margarita Mishina, Elena Petricci, Yves Pommier, Karin C. Nitiss, John L. Nitiss

**Affiliations:** ^1^ Pharmaceutical Sciences Department, University of Illinois College of Pharmacy, Rockford, IL, United States; ^2^ Laboratory of Molecular Pharmacology, Developmental Therapeutics Branch, Center for Cancer Research, National Cancer Institute, National Institutes of Health, Bethesda, MD, United States; ^3^ St. Jude Children’s Research Hospital Memphis, Memphis, TN, United States; ^4^ Universita’ degli Studi di Siena, Siena, Italy

**Keywords:** Mre11-Rad50-Nbs1 complex, topoisomerase II (TOP2), proteasome, VCP/p97, DNA-protein crosslink (DPC) repair

## Abstract

During a normal topoisomerase II (TOP2) reaction, the enzyme forms a covalent enzyme DNA intermediate consisting of a 5′ phosphotyrosyl linkage between the enzyme and DNA. While the enzyme typically rejoins the transient breakage after strand passage, a variety of conditions including drugs targeting TOP2 can inhibit DNA resealing, leading to enzyme-mediated DNA damage. A critical aspect of the repair of TOP2-mediated damage is the removal of the TOP2 protein covalently bound to DNA. While proteolysis plays a role in repairing this damage, nucleolytic enzymes must remove the phosphotyrosyl-linked peptide bound to DNA. The MRN complex has been shown to participate in the removal of TOP2 protein from DNA following cellular treatment with TOP2 poisons. In this report we used an optimized ICE (*In vivo* Complex of Enzyme) assay to measure covalent TOP2/DNA complexes. In agreement with previous independent reports, we find that the absence or inhibition of the MRE11 endonuclease results in elevated levels of both TOP2α and TOP2β covalent complexes. We also examined levels of TOP2 covalent complexes in cells treated with the proteasome inhibitor MG132. Although MRE11 inhibition plus MG132 was not synergistic in etoposide-treated cells, ectopic overexpression of MRE11 resulted in removal of TOP2 even in the presence of MG132. We also found that VCP/p97 inhibition led to elevated TOP2 covalent complexes and prevented the removal of TOP2 covalent complexes by MRE11 overexpression. Our results demonstrate the existence of multiple pathways for proteolytic processing of TOP2 prior to nucleolytic processing, and that MRE11 can process TOP2 covalent complexes even when the proteasome is inhibited. The interactions between VCP/p97 and proteolytic processing of TOP2 covalent complexes merit additional investigation.

## Introduction

Type II topoisomerases (TOP2) resolve topological issues that arise during DNA metabolic events such as replication, transcription and chromosomal segregation by cleaving both strands of DNA, carrying out strand passage, and subsequent resealing of the DNA (Nitiss, 2009a; Vos et al., 2011; Chen et al., 2013; Pommier et al., 2022). During the breaking-rejoining catalytic cycle, TOP2 breaks the phosphodiester DNA backbone by a transesterification reaction, leading to covalent attachment of the protein to DNA by a 5′ phosphotyrosyl bond. This mechanism allows facile resealing by transesterification to regenerate the phosphodiester backbone of DNA and release the bound topoisomerase (Wang, 1998; Pommier et al., 2022). The TOP2 reaction appears to have high fidelity, in the sense that the enzyme rarely becomes covalently trapped on DNA due to a failure to carry out the re-ligation reaction (Nitiss et al., 2019). However, recent results have suggested that the TOP2β isoform of TOP2 found in vertebrates may form long-lasting TOP2/DNA complexes (Ju et al., 2006; Haffner et al., 2010; Pommier et al., 2016; Puc et al., 2017). The rejoining reaction can also be disrupted by small molecule inhibitors of the reaction (Li and Liu, 2001); these small molecules have found clinical uses as both antibacterial and anticancer agents (Nitiss, 2009b; Drlica et al., 2009; McKie et al., 2021). Structural alterations in DNA, including DNA damage and non B-DNA structures, can also alter the enzyme cleavage/resealing cycle (Wilstermann and Osheroff, 2001; Nitiss et al., 2019; Zell et al., 2021; Pommier et al., 2022).

TOP2-induced DNA damage may lead to both cytotoxicity and genome instability (Canela et al., 2019; Pommier et al., 2022). The genome instability induced by topoisomerase inhibition or misfunction may have dire consequences including malignancies (Pendleton et al., 2014; Boot et al., 2022). Therefore, cells have a diverse repertoire of DNA repair and damage tolerance mechanisms to prevent topoisomerase-induced genome instability (Weickert and Stingele, 2022). These various pathways include DNA damage signaling, removal of proteins bound to DNA by proteolysis (Li and Liu, 2001), and a variety of nucleolytic enzymes that can excise phosphotyrosyl-linked peptides (Yang et al., 1996; Cortes Ledesma et al., 2009) or carry out endonucleolytic cleavage to remove DNA/protein crosslinks (Swan et al., 2022)

MRE11 is a double strand break (DSB) repair enzyme with endonuclease and 3′ to 5′ exonuclease activities, acting in a functional protein complex with RAD50 and NBS1. The MRE11-RAD50-NBS1 (MRN) complex plays a central role in sensing and initial processing of DSB termini to promote HR or NHEJ-mediated repair (Paull, 2018). Genetic studies on yeast meiotic recombination indicated that SPO11, a eukaryotic protein related to type VI topoisomerases, generates DNA breaks by a topoisomerase-like mechanism (Keeney et al., 1997), and that disjoining of SPO11 covalently bound to DNA requires MRE11 complexes (Keeney et al., 2014). An additional key endonucleolytic protein that functions with the MRN complex is CtIP (*SAE2* in *S. cerevisiae*; encoded by RBBP8 in humans) which is also required for disjoining SPO11 during meiotic recombination (Makharashvili and Paull, 2015).

The roles of MRE11 and CtIP in meiotic recombination, specifically the requirement for both MRE11 and CtIP (Sae2) to remove a protein covalently bound to DNA by a phosphotyrosyl linkage suggested that these proteins could also participate in removing topoisomerases covalently trapped on DNA. Experiments in yeast first provided more direct support for a role for the Mre11 complexes in removing Spo11 along with some evidence for the complex in processing Top2 covalent complexes formed in the presence of etoposide (Neale et al., 2005). These results were further expanded in fission yeast and showed roles for MRE11 and CtIP in processing damage arising from small molecules that trap topoisomerases onto DNA. Also of note, Hartsuiker and colleagues showed that both Top1 and Top2 could be processed by Mre11 and CtIP (Hartsuiker et al., 2009). Subsequent work in mammalian cells lent support for a role for MRE11 in processing trapped TOP2 covalent complexes (Lee et al., 2012; Aparicio et al., 2016); and Lee and colleagues suggested a preference for processing the TOP2α isoform, a result not seen in subsequent work. These studies were further expanded by Hoa and colleagues (Hoa et al., 2016) who suggested that TOP2 failure, as determined by levels of TOP2 covalent complexes could be readily observed in cells lacking MRE11 even in the absence of small molecule inhibitors, suggesting that the considerations described above such as trapping of TOP2 by DNA damage or non-B-DNA structures are significant under normal cell growth conditions. The authors suggested that repair of TOP2-induced damage is likely a contributing factor to the essentiality of MRE11 (Xiao and Weaver, 1997; Stewart et al., 1999; Buis et al., 2008). Subsequent work has highlighted the importance of the NBS1 component in processing DNA breaks with blocked termini (Deshpande et al., 2016), and the importance of processing for subsequent strand break repair (Nakamura et al., 2010).

Despite the now extensive literature on the role of MRE11 in processing damage, several important questions remain unanswered. Of particular interest are questions relating to recognition of a trapped TOP2 enzyme as opposed to an enzyme undergoing a normal catalytic cycle. To answer these questions, we developed a robust mammalian cell system that could be used to address the coordination of MRE11 with other pathways that may be prerequisites for normal processing by MRE11. It is noteworthy that processing by nucleolytic pathways such as those mediated by TDP1 or TDP2 typically require proteolysis before nucleolytic removal of the trapped protein (Interthal and Champoux, 2011; Gao et al., 2014), although accessory proteins have been recently described to eliminate the need for proteolysis prior to nucleolytic removal (Schellenberg et al., 2017; Zagnoli-Vieira and Caldecott, 2017).

In the first part of this study, we establish a simple system for assessing the repair of topoisomerase-induced damage using the ICE assay originally developed by Muller and colleagues (Subramanian et al., 1998) and subsequently refined in our laboratory (Anand et al., 2018). The guiding hypothesis of this assay is that a defect in removing TOP2 protein trapped on DNA by small molecules or other processes will lead to elevated levels of TOP2 covalent complexes (TOP2ccs) compared to control cells. After showing that this assay appropriately reports the roles of MRE11, NBS1 and CtIP in leading to elevated levels of TOP2cc for both TOP2α and TOP2β, we demonstrate that the MRE11 endonuclease activity is responsible for the repair reaction and demonstrate that previously described inhibitors are specific for MRE11 endonuclease, rather than other putative nuclease activities. Finally, we show that MRE11 can process TOP2ccs without proteolysis, but that under conditions of normal levels of expression, MRE11 has limited activity against TOP2ccs.

## Results

### MRE11 depletion enhances TOP2α- and TOP2β-DNA covalent complexes

**FIGURE 1:**
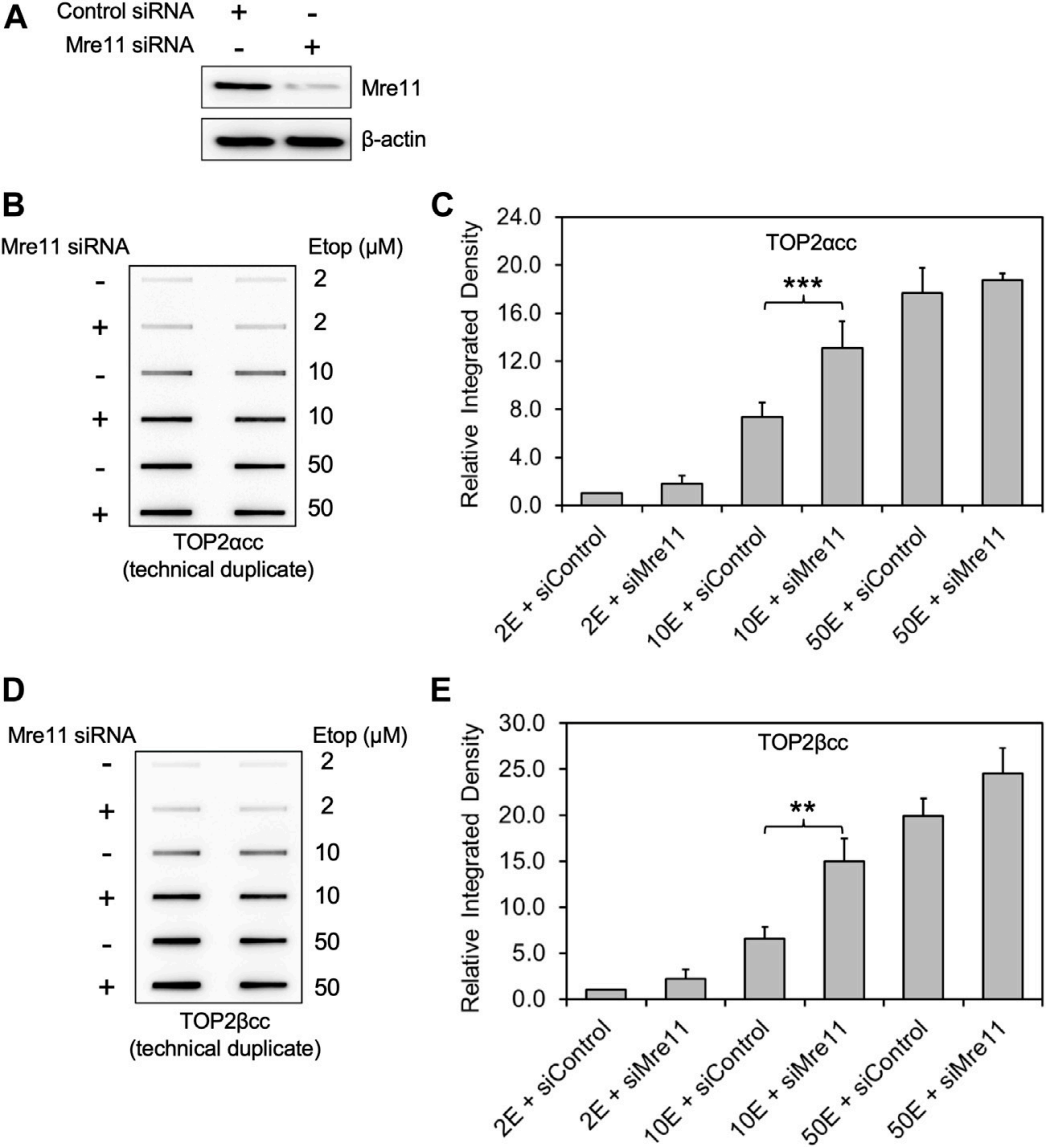
Knockdown of MRE11 by siRNA increases the level of etoposide-induced TOP2-DNA complexes as detected by ICE. RH30 cells were transfected with non-targeting siRNA control (siControl) or siRNA targeting MRE11 (siMRE11), followed by treatment with etoposide (Etop) at the indicated concentrations. **(A)** Western blot assessing MRE11 knockdown efficiency. Semi-quantitative image analysis of immunoblots using ImageJ indicates that treatment with siMre11 resulted in 94% knockdown of endogenous MRE11 (quantitation not shown). **(B)** ICE assay illustrating TOP2αcc in MRE11 knockdown cells. The panel illustrates a representative assay. In all cases, the assay includes technical replicates. At least three biological replicates were performed for all experimental conditions. No detectable signal is ever observed under our conditions in cells treated with a solvent control, over more than 20 experiments. Samples without etoposide were therefore not typically analyzed in ICE assays. **(C)** Densitometric analysis of all experiments comparing relative integrated densities of TOP2αcc signal amongst MRE11 knockdown and control cells treated with 2 μM, 10 and 50 μM etoposide (2E, 10E and 50E). Integrated density of TOP2αcc signal of each group was normalized to that of control cells treated with 2 μM etoposide. *** denotes *p*-value < 0.001, determined using Student’s t-test. Samples not marked with a bar did not result in a statistically significant difference. **(D)** ICE assay illustrating TOP2βcc. The panel illustrates a representative assay. In all cases, the assay includes technical replicates. **(E)** Densitometric analysis of all experiments comparing relative integrated densities of TOP2βcc signal amongst MRE11 knockdown and control cells treated with 2 μM, 10 and 50 μM etoposide. Integrated density of TOP2αcc signal of each group was normalized to that of control cells treated with 2 μM etoposide. ** denotes *p*-value < 0.01.

We examined the accumulation of trapped TOP2 using the ICE bioassay (Anand et al., 2018; Nitiss et al., 2021)by transiently transfecting RH30 cells, a pediatric rhabdomyosarcoma cell line with siRNA against MRE11 or non-targeting siRNA (Figure 1A) followed by treatment with etoposide at various concentrations (2, 10, 50 μM) for 2 h. In accord with findings that MRN complex is involved in the release of the TOP2-like protein Spo11 from DNA in *S. cerevisiae* and that Rad32 (Mre11) plays a role in Top2 removal in *S. pombe* (Hartsuiker et al., 2009), we found that both hTOP2α and βcc levels in MRE11-deficient cells were distinctly higher than those in WT cells (transfected with control siRNA) treated with 10 μM etoposide (Figure 1B). However, for etoposide treatments at low concentration (2 μM) or high concentration (50 μM), we failed to observe a significant difference in TOP2α or TOP2βcc levels between MRE11 knockdown cells and WT cells.

To quantitate the TOP2cc levels, we performed densitometric analysis using ImageJ and determined relative integrated densities of TOP2cc signal amongst all groups. We found that, when treated with 10 μM etoposide, MRE11-depleted cells showed significantly increased levels of TOP2αcc compared to WT cells (Figure 1C; 1.77 ± 0.03-fold increase after 10 μM etoposide treatment; *p* = 0.0003, *n* = 3). Like our data on TOP2αcc detection, MRE11 knockdown resulted in an increased accumulation of TOP2βcc compared to WT cells when we treated the cells with 10 μM etoposide (Figures 1D,E; 2.29 ± 0.19-fold increase after 10 μM etoposide treatment; *p* = 0.008, *n* = 3). These results demonstrate that MRE11 reduces etoposide-induced TOP2ccs but that its activity is insufficient at high etoposide concentrations.

### NBS1 depletion enhances TOP2α- and TOP2β-DNA covalent complexes

NBS1 is a key component of the MRN complex that interacts with MRE11 and RAD50 through its C-terminal binding domain and recruits MRN to DSB sites by direct binding to phosphorylated histone H2AX (Paull, 2018) upon its phosphorylation by ATM in response to DNA damage. Recent studies show that several enzymatic activities of the MRN complex including MRE11-mediated DSB end-processing are lost in the absence of NBS1 (Deshpande et al., 2016), suggesting a key role of NBS1 in regulating MRN complex function.

**FIGURE 2:**
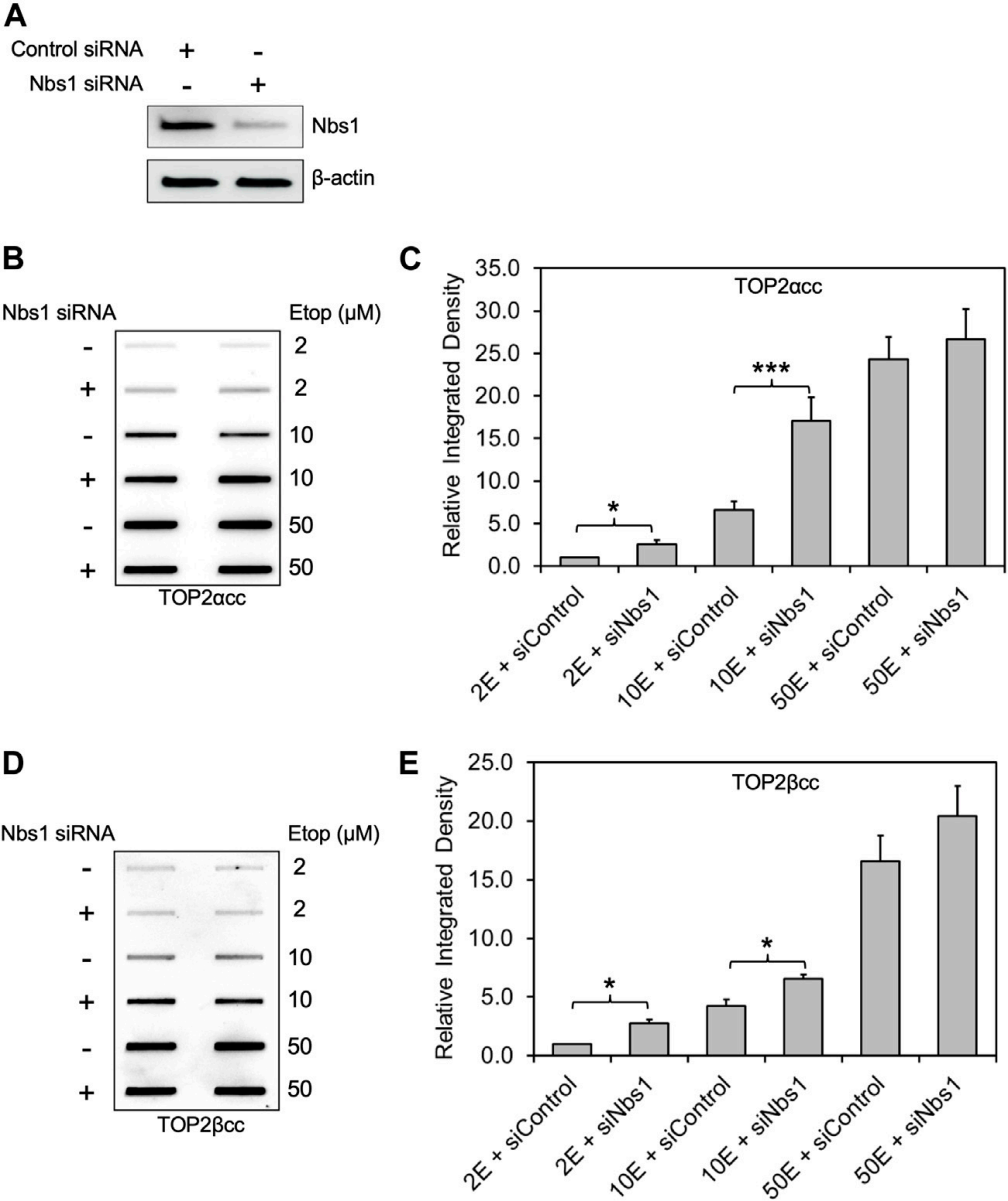
Knockdown of NBS1 by siRNA increases the level of etoposide-induced TOP2-DNA complexes. Experimental conditions and methods were the same as Figure 1. **(A)** Western blot assessing NBS1 levels using antibody against NBS1. Semi-quantitative image analysis of immunoblots using ImageJ indicated that treatment with siNbs1 resulted in 85% knockdown of endogenous NBS1 (quantitation not shown). β-actin was used as a protein loading control. **(B)** ICE assay illustrating TOP2αcc in NBS1 knockdown cells. The panel illustrates a representative assay. **(C)** Densitometric analysis of all experiments comparing relative integrated densities of TOP2αcc signal amongst NBS1 knockdown and control cells treated with 2 μM, 10 and 50 μM etoposide. Integrated density of TOP2αcc signal of each group was normalized to that of control cells treated with 2 μM etoposide. * denotes *p*-value <0.05. Samples not marked with a bar did not result in a statistically significant difference. **(D)** ICE assay illustrating TOP2βcc. The panel illustrates a representative assay. **(E)** Densitometric analysis of all experiments comparing relative integrated densities of TOP2βcc signal amongst MRE11 knockdown and control cells.

For these reasons, we assessed the role of NBS1 in TOP2cc removal by knocking-down endogenous NBS1 in RH30 cells. After NBS1 siRNA transfection (Figure 2A), etoposide treatment with increasing concentrations (2, 10 and 50 μM) showed a significant increase in both TOP2α and TOP2βcc levels in NBS1-deficient cells compared to WT cells at 2 h after addition of 10 μM etoposide. Upon exposure to high concentration (50 μM) of etoposide, NBS1 deficient cells did not generate significantly higher levels of TOP2αcc or TOP2βcc than did WT cells, consistent with our observation in MRE11 knockdown cells (Figures 2B–E). In slight contrast to our finding with treatment in MRE11-knockdown cells with low concentration (2 μM) of etoposide, we observed a significant elevation in both TOP2α and TOP2βcc levels in NBS1-knockdown cells. Since the levels of covalent complexes at 2 μM etoposide were similar in the knockdown of MRE11 or NBS1, our results do not suggest a significant difference between loss of MRE11 or knockdown of NBS1. Thus, our results agree with previous work on the role of NBS1 as a component of the MRN complex in repairing TOP2-DNA covalent complexes (Deshpande et al., 2016).

### CtIP depletion and MRE11 depletion are epistatic for the processing of TOP2α/β-DNA covalent complexes

**FIGURE 3:**
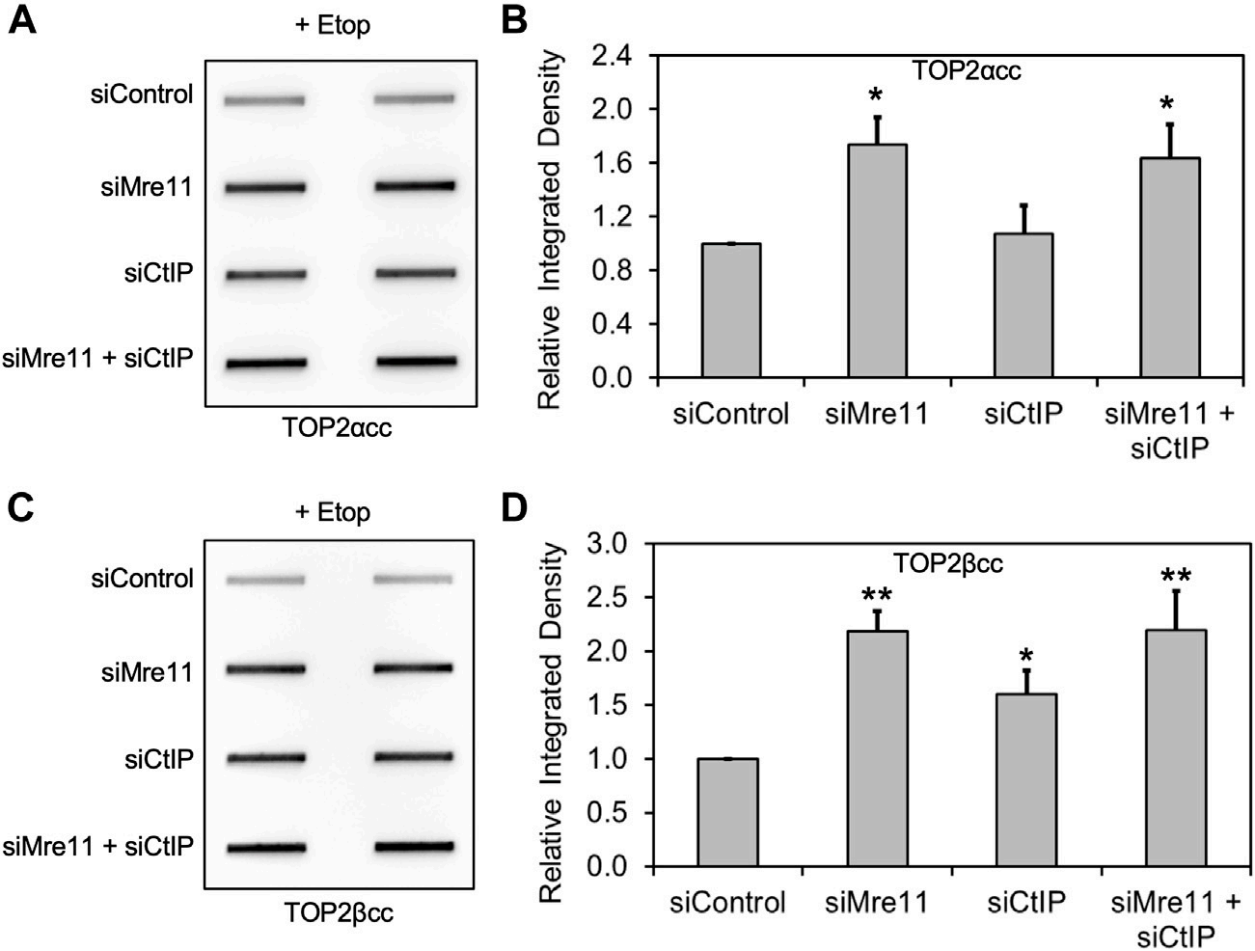
Knockdown of MRE11 and CtIP are epistatic for repair of TOP2 covalent complexes. We carried out siRNA knockdowns as in Figure 1 (for MRE11) and Supplementary Figure S1 (for CtIP). The results shown were following treatment with 10 μM etoposide for 2 h. **(A)** ICE assay illustrating TOP2αcc under the conditions indicated. **(B)** Densitometric analysis of all experiments comparing relative integrated densities of TOP2αcc signal. Integrated density of TOP2αcc signal of each group was normalized to that of cells treated with siControl. **(C)** ICE assay illustrating TOP2βcc under the conditions indicated. **(D)** Densitometric analysis of all experiments comparing relative integrated densities of TOP2βcc signal.

Because CtIP functions in concert with MRE11 nuclease activity (Makharashvili and Paull, 2015), we carried out a similar series of experiments with CtIP, using siRNA knockdown in RH30 cells. As seen with MRE11 and NBS1 knockdowns, CtIP depletion also led to elevated levels of TOP2 covalent complexes (Supplementary Figures S1A–E). To demonstrate whether CtIP was working in coordination with the MRN complex, we simultaneously knocked down both CtIP and MRE11. While both single knockdowns led to elevated TOP2ccs, simultaneous knockdown of both proteins did not result in higher TOP2cc levels than knocking down MRE11 alone (Figures 3A–D). The results in both Figure 3 and Supplementary Figure S1 showed modest effects on TOP2ccs, especially with TOP2αcc. The relatively inefficient knockdown of CtIP may be due to its ability to regulate its own expression (Mozaffari et al., 2021).

### The endonuclease activity of MRE11 is required for the processing TOP2α/β-DNA covalent complexes

**FIGURE 4:**
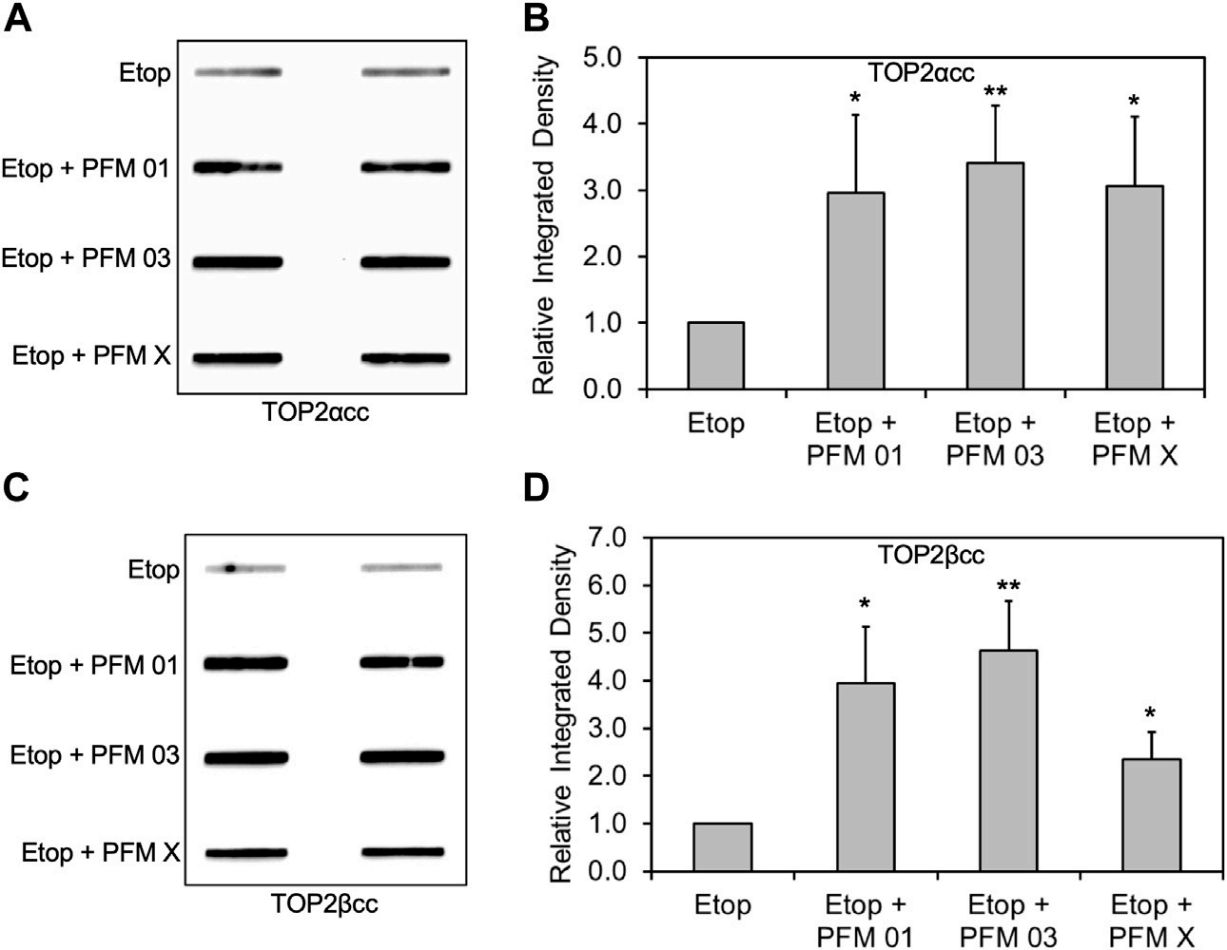
Inhibition of MRE11 endonuclease activity results in accumulation of etoposide-induced TOP2/DNA complexes. RH30 cells were pre-treated with 25 μM PFM01, 25 μM PFM03, or 25 μM PFM X for 4 h. Etoposide (10 μM) was added, and incubation was continued for 2 h. Cells were then harvested for ICE assays. **(A)** Representative ICE assay measuring the levels of TOP2αcc **(B)** Densitometric analysis of all assays comparing relative integrated densities of TOP2αcc signals. Integrated density of TOP2αcc signal of each group was normalized to that of cells treated with etoposide alone. **(C)** Representative ICE assay measuring the levels of TOP2βcc. **(D)** Densitometric analysis of all assays comparing relative integrated densities of TOP2βcc signals. Integrated density of TOP2βcc signal of each group was normalized to that of cells treated with etoposide alone.

MIRIN was discovered as a small molecule inhibitor of the MRN complex (Dupre et al., 2008), and more recently, several derivatives with specificity toward the endo or exonuclease activity of MRE11 have been reported (Shibata et al., 2014). We employed three specific MRE11 endo- and endo/exonuclease inhibitors to demonstrate the roles of specific MRE11 nuclease activities. RH30 cells were pre-exposed to 25 μM PFM01 (MRE11 endonuclease inhibitor), 25 μM PFM03 (MRE11 endonuclease inhibitor), or 25 μM PFMX (MRE11 endo/exonuclease inhibitor) for 4 h, followed by treatment with etoposide for an additional 2 h. Chemical inhibition of MRE11 endonuclease activity in combination with etoposide treatment increased the levels of both TOP2α and TOP2βcc in RH30 cells compared to etoposide treatment alone (Figures 4A,C). Densitometric analysis shows that etoposide in combination with PFM01, PFM03 or PFMX led to an increase in the total amount of covalently bound TOP2α and TOP2β compared to treatment with etoposide alone (Figures 4B,D). By contrast, treatment using the same conditions with the MRE11 exonuclease inhibitor PFM39 and etoposide failed to elicit higher levels of TOP2ccs than treatment with etoposide alone (Supplementary Figures 2A–D).

**FIGURE 5:**
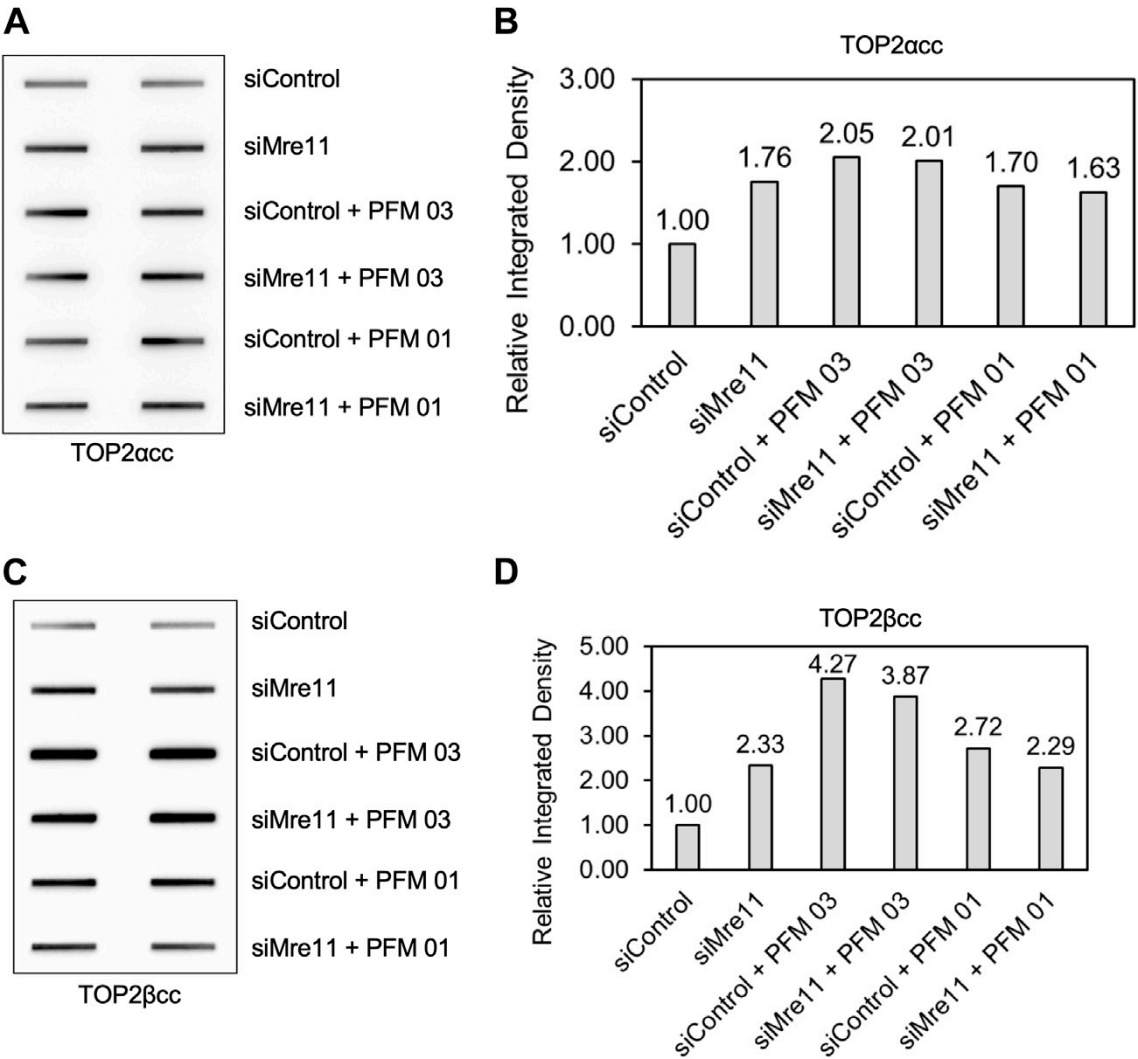
Inhibition of MRE11 endonuclease activity does not change the level of TOP2 covalent complexes when MRE11 is depleted. RH30 cells were transfected with non-targeting siRNA control (siControl) or siRNA targeting MRE11 (siMRE11) as in Figure 1. These cells were then pre-treated with 25 μM PFM03 where indicated. Etoposide (10 μM) was added, and incubation was continued for 2 h before ICE assays. **(A)** Representative ICE assay measuring the levels of TOP2αcc. **(B)** Densitometric analysis of all assays comparing relative integrated densities of TOP2αcc signals. Integrated density of TOP2αcc signal of each group was normalized to that of siControl cells treated with etoposide alone. Levels of TOP2αcc for siControl versus siMre11 cells treated with PFM03 were not statistically different. **(C)** Representative ICE assay measuring the levels of TOP2βcc. **(D)** Densitometric analysis of all assays comparing relative integrated densities of TOP2βcc signals.

We next assessed the specificity of the MRE11 inhibitors by treating RH30 cells that had been pretreated with siRNA directed against MRE11 and then follow up with treatment with PFM01 or PFM03. Concurrent treatment of cells with knocked down MRE11, etoposide, and the small molecule inhibitor did not result in a difference in levels of TOP2α or TOP2βcc compared to treatment with a control siRNA, etoposide, and the small molecule inhibitors (Figures 5A–D). This indicates that MRE11 must be present in order for the small molecules to impact TOP2cc levels, indicating that these small molecules do not affect other targets that impact repair of TOP2-induced damage.

### Combination of MRE11 inhibition and proteasome inhibition does not lead to additive increase in TOP2cc levels

**FIGURE 6:**
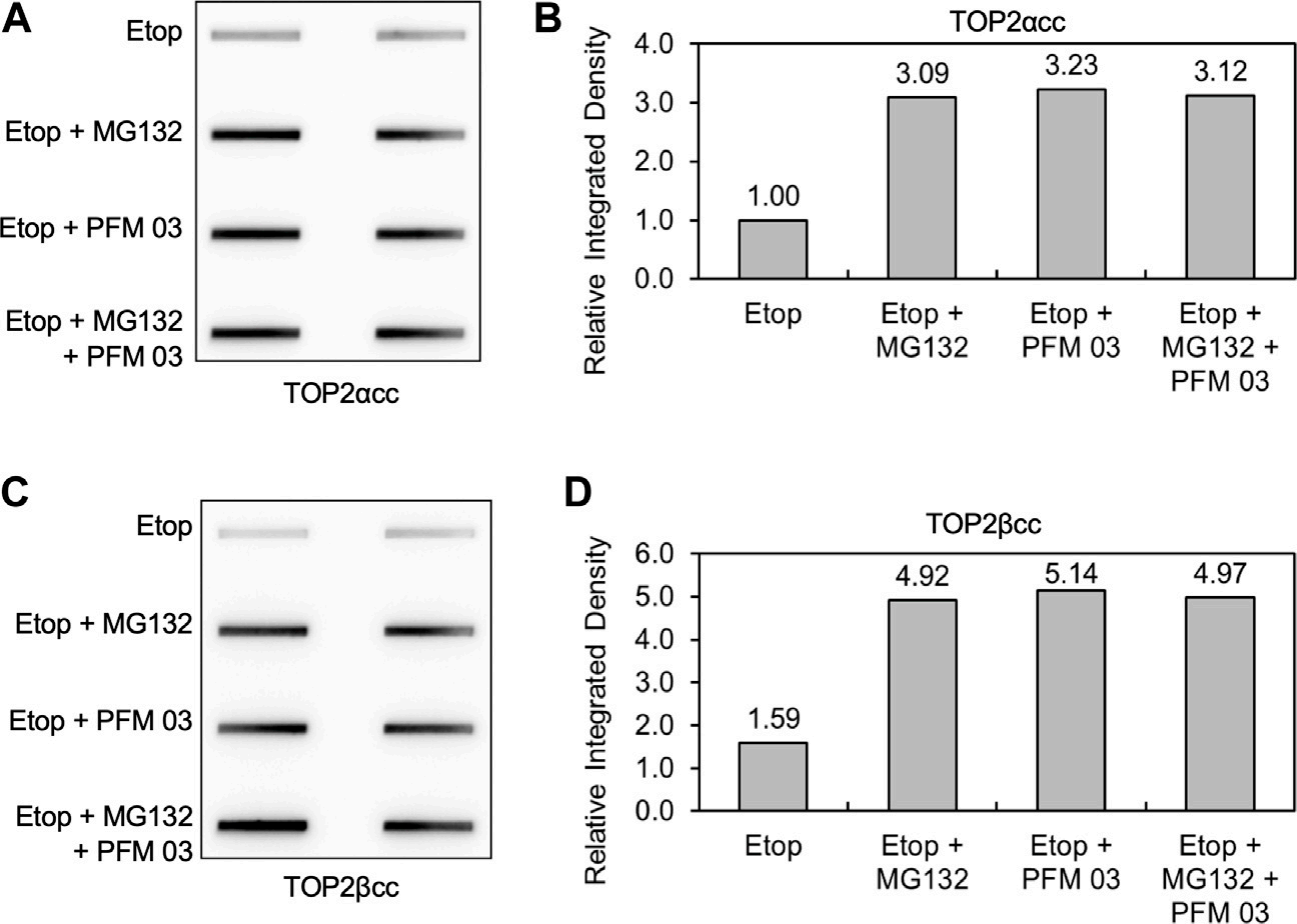
MRE11 inhibitors do not enhance levels of covalent complexes upon concurrent treatment with proteasome inhibitors. RH30 cells were pre-treated with 25 μM PFM03, 10 μM MG132, or 25 μM PFM 03 + 10 μM MG132 for 4 h. Etoposide (10 μM) was added, and incubation was continued for 2 h before ICE assays. **(A)** Representative ICE assay measuring the levels of TOP2αcc. **(B)** Densitometric analysis of all assays comparing relative integrated densities of TOP2αcc signals. Integrated density of TOP2αcc signal of each group was normalized to that of cells treated with etoposide alone. **(C)** Representative ICE assay measuring the levels of TOP2βcc. **(D)** Densitometric analysis of all assays comparing relative integrated densities of TOP2βcc signals.

We next assessed the importance of the proteasome in the ability of MRE11 to disjoin TOP2ccs. As noted in the introduction, removal of topoisomerase covalent complexes frequently requires prior proteolysis as tyrosyl DNA phosphodiesterases have limited activity against full-length topoisomerases covalently bound to DNA (Sun et al., 2020a; Sun et al., 2020b; Sun et al., 2020c). After pretreatment with MG132, PFM03 or the combination, RH30 cells were treated with etoposide for 2 h, and the levels of covalent complexes were assessed by the ICE assay (Figures 6A–D). As previously shown, MG132 led to elevated levels of TOP2ccs upon etoposide treatment (Sun et al., 2022a). The level of TOP2ccs for both TOP2α and TOP2βccs upon treatment with etoposide and PFM03 was also elevated compared to etoposide alone. However, the combination of MG132 plus PFM03 did not yield a further increase in Top2ccs, indicating that MRE11 is epistatic to the proteasome for the removal of TOP2ccs induced by etoposide. The simplest interpretation is that the proteasome is required for processing of TOP2ccs by the MRE11 endonuclease activity. However, this conclusion requires modification as noted in the following section.

### Upregulation of MRE11 leads to processing of TOP2ccs in the presence of proteasome inhibitor

**FIGURE 7:**
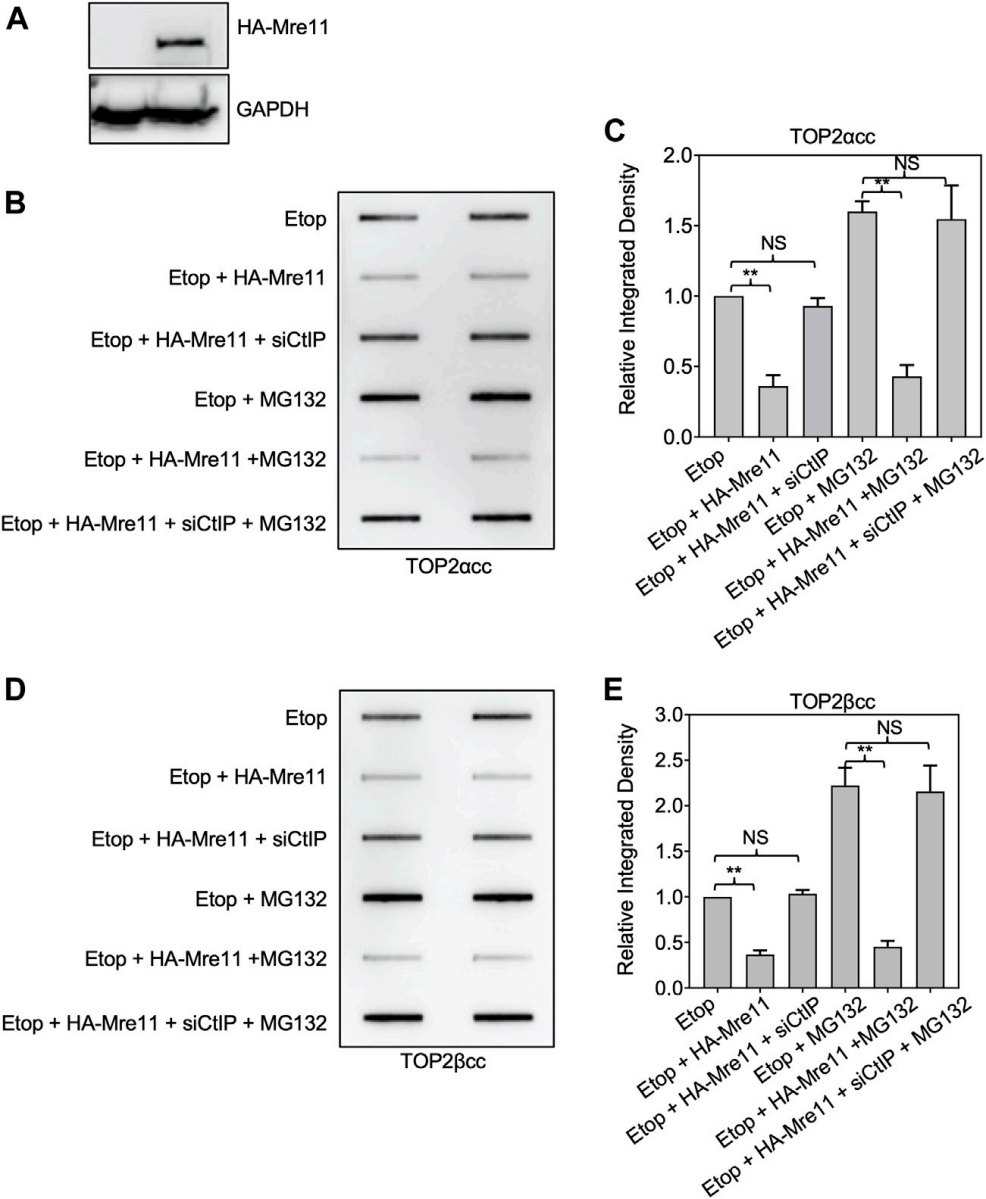
Ectopic expression of MRE11 results in removal of TOP2ccs in cells treated with proteasome inhibitors. HEK293 cells were transfected with indicated plasmid and or siRNA for 48 h prior to treatment with 10 μM MG132 for 1 h. Etoposide (10 μM) was added, and incubation was continued for 1 h before ICE assays. **(A)** Western blot assessing MRE11-HA plasmid transfection efficiency. **(B)** Representative ICE assay measuring the levels of TOP2αcc. **(C)** Densitometric analysis of all assays comparing relative integrated densities of TOP2αcc signals of cells. Integrated density of TOP2αcc signal of each group was normalized to that of control cells (no transfection) treated with etoposide alone. **(D)** Representative ICE assay measuring the levels of TOP2βcc. **(E)** Densitometric analysis of all assays comparing relative integrated densities of TOP2βcc signals.

To further assess whether proteasome activity is required for MRE11-dependent processing, we used a plasmid for ectopic overexpression of MRE11. This plasmid expressing HA-tagged MRE11 was transfected into HEK293 cells (Figure 7A). Cells expressing the ectopically expressed MRE11 exhibited reduced levels of etoposide-induced TOP2ccs (Figures 7B–E). Notably, CtIP was still required since siRNA directed against CtIP prevented the MRE11-induced processing. As shown above, treatment with MG132 led to elevated levels of TOP2ccs compared to treatment with etoposide alone. Cells expressing HA-MRE11 showed the same reduction in TOP2 covalent complexes in etoposide plus MG132 treated cells as cells treated with etoposide alone. This result complements those shown in Figure 6 for the combination of the MRE11 endonuclease inhibitor and MG132 and suggests that MRE11 overexpression is sufficient to overcome the requirement of the proteasome for TOP2cc processing under physiological levels of MRE11. Of note, Figure 7 shows that similar results are seen with both TOP2α and TOP2βccs.

### MRE11 upregulation fails to overcome VCP/p97 inhibition for TOP2cc processing

VCP/p97 is an AAA + protein that enhances proteolysis of selected substrates both from cell membranes and within the nucleus (Meyer and Weihl, 2014). Austin and colleagues recently reported that VCP/p97 inhibition hinders the repair of TOP2ccs (Swan et al., 2021). Similar to their results, we found that the VCP/p97 inhibitor NMS873 (Magnaghi et al., 2013) plus etoposide resulted in elevated levels of TOP2ccs compared to etoposide alone (Supplementary Figures 3A–D). We also found that co-treatment with etoposide plus MG132 and NMS873 showed epistasis with etoposide and MG132, leading to higher levels of TOP2ccs than treatment with etoposide and NMS873. Etoposide plus MG132 and NMS873 led to the same levels of TOP2ccs as treatment with etoposide and MG132. These results are consistent with a model of enhanced degradation of TOP2ccs mediated by VCP/p97 action and would place VCP/p97 upstream of proteasomal degradation.

**FIGURE 8:**
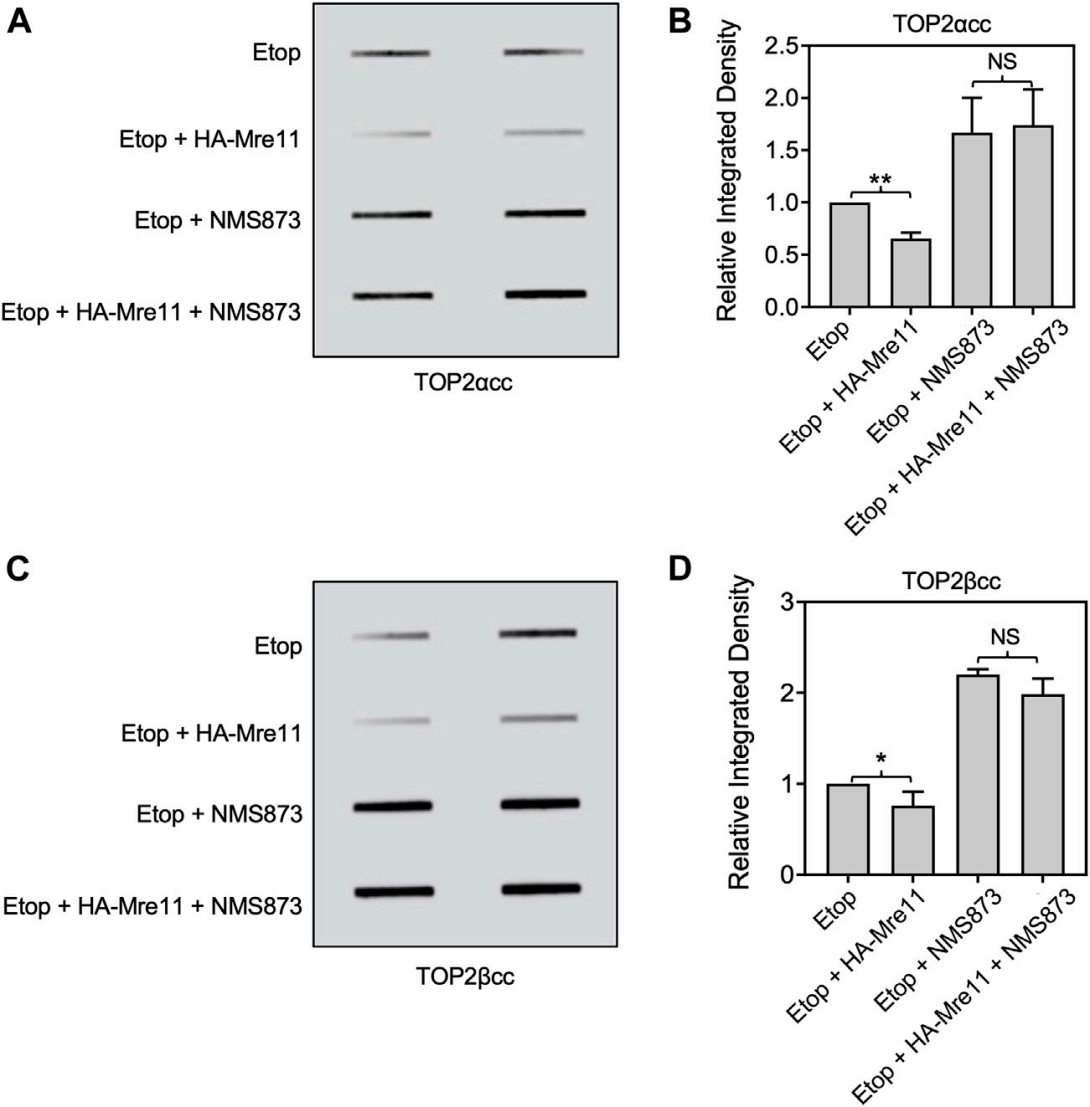
Inhibition of VCP/p97 prevents processing by ectopic expression of MRE11. HEK293 cells were transfected with HA-MRE11 overexpressing plasmid for 48 h prior to treatment with 10 μM NMS873 1 h. Etoposide (10 μM) was added, and incubation was continued for 1 h before ICE assays. **(A)** Representative ICE assay measuring the levels of TOP2αcc. **(B)** Densitometric analysis of all assays comparing relative integrated densities of TOP2αcc signals. Integrated density of TOP2αcc signal of each group was normalized to that of control cells (no transfection) treated with etoposide alone. **(C)** Representative ICE assay measuring the levels of TOP2βcc. **(D)** Densitometric analysis of all assays comparing relative integrated densities of TOP2βcc signals. NS, not significant.

We assessed the effect of VCP/p97 inhibition by NMS873 on MRE11-dependent processing using ectopic expression of MRE11 as described above. In contrast to the results shown above with MG132, treatment of cells with NMS873 prevented MRE11-dependent loss of TOP2ccs (Figures 8A–D). This result suggests that processing of TOP2ccs is essential for their removal by an MRE11-dependent pathway and taken with the results from Figures 6, 7 suggests that the normal pathway of MRE11-dependent removal of TOP2ccs involves processing by VCP/p97 and proteolysis. Lack of proteolysis can be overcome by MRE11 overexpression, but VCP/p97 processing is essential.

### A novel substrate system for assessing TOP2cc repair *in vitro*

**FIGURE 9:**
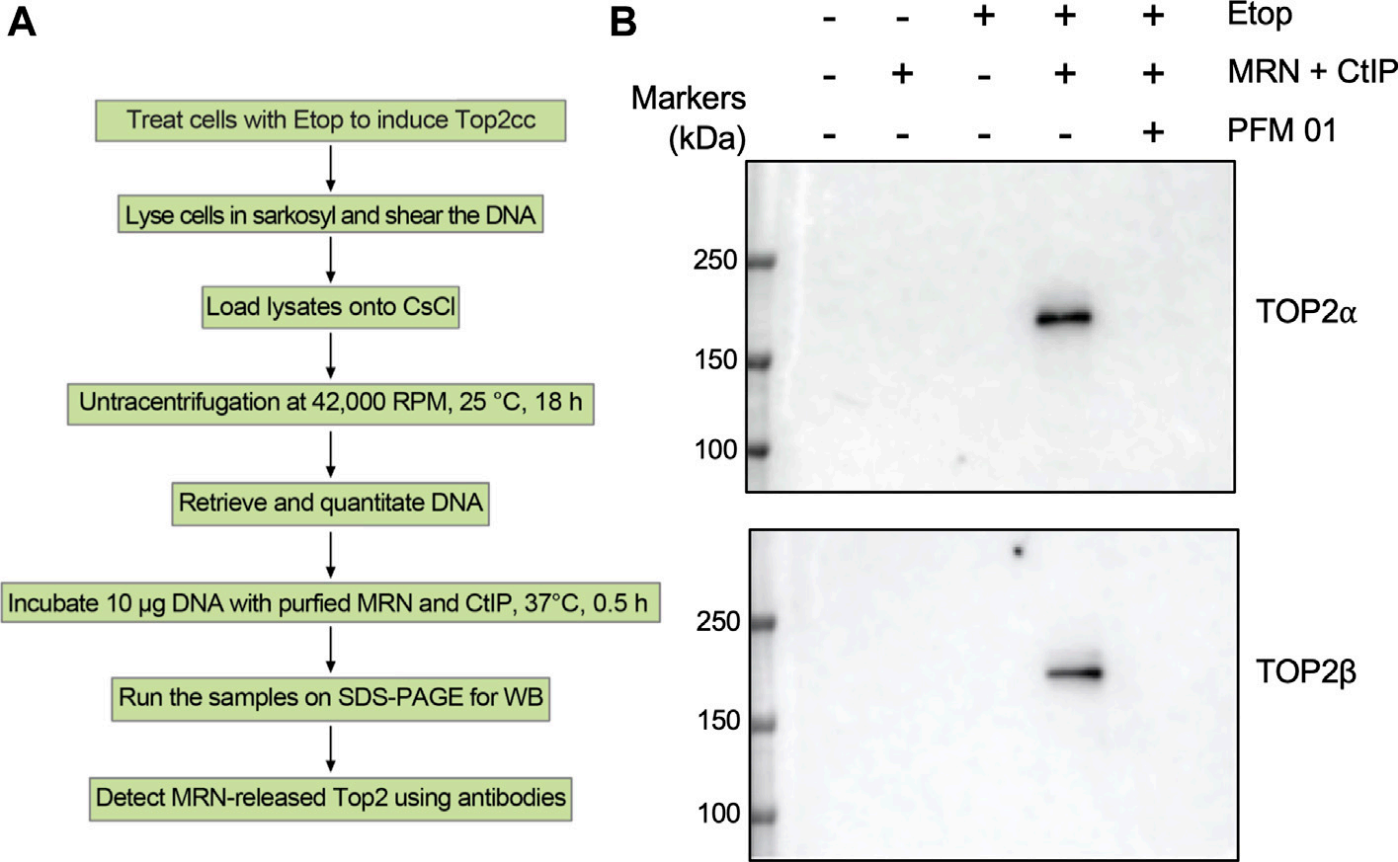
*In vitro* processing of TOP2 covalent complexes by purified MRN proteins. **(A)** Scheme for *in vitro* TOP2cc repair assay using ICE samples. **(B)** HEK293 cells were treated with Etoposide (10 μM) for 30 min and then subjected to ICE assays to induce TOP2cc. 10 µg DNA samples isolated by ICE assay were incubated with purified human MRN complex (100 nM) and CtIP (100 nM) for 30 min at 37°C, followed by addition of Laemmli buffer and Western blotting.

The experiments above have all taken advantage of *in vivo* systems that report levels of TOP2 covalent complexes under various conditions. Devising substrates for repair of TOP2 covalent complexes *in vitro* has been challenging, and substrates that can be synthesized readily often have unphysiological characteristics such as unique DNA sequences and single strand/double strand DNA junctions (Nitiss et al., 2006). We reasoned that the TOP2ccs purified on cesium gradients represented an ideal substrate for *in vitro* TOP2cc repair assays. DNA purified from etoposide-treated cells (which therefore contain TOP2ccs) was used as a substrate for reactions with MRN components. Figure 9 shows the overall experimental approach (Figure 9A) and the results obtained following treatment with purified MRN and CtIP proteins. In brief, HEK293 cells were treated with 200 µM etoposide for 30 min, and lysates were purified on CsCl gradients. Recovered DNA was quantitated, and 10 μg of DNA was added to reactions that included purified MRN (a generous gift of T. Paull, UT Austin). Control extracts prepared from cells that were not exposed to etoposide generated no liberated TOP2 as expected since there is barely any endogenously trapped TOP2cc in the absence of TOP2 poison. Addition of MRN plus CtIP to DNA from etoposide-treated cells yielded free TOP2α and TOP2β, as detected by Western analysis of treated samples (Figure 9B). Pre-incubation of purified MRN with PFM01 for 10 min did not yield free TOP2α and TOP2β, indicating that MRE11 endonuclease activity is required for processing. Taken together, our results show that DNA purified from etoposide-treated cells can be used as a substrate for enzyme activities that can disjoin TOP2/DNA covalent complexes. The preparation of ICE samples includes treatment with Sarkosyl and ultracentrifuge with CsCl, which denature TOP2. Therefore, this experiment does not imply that the MRN complex can process native (undenatured) TOP2ccs.

## Discussion

Topoisomerases are critical for genome stability, and in their absence, DNA metabolic activities lead to structures such as R-loops that compromise genome stability. Yet, the topoisomerase reaction is a risky undertaking, since altering DNA topology absolutely requires (transient) DNA breakage. Failure of the topoisomerase reaction is a unique issue since the abortive enzyme reaction intermediate is potentially toxic and genome destabilizing (Pommier et al., 2022). The MRN complex is a key player in genome stability, and our work, and the studies described in the introduction highlight its importance in protecting cells from topoisomerase-induced DNA damage. The recognition of topoisomerase-induced damage remains a largely unanswered question, since a topoisomerase trapped as a cleavage complex is difficult to distinguish from an enzyme carrying out its normal reaction. Understanding the detailed pathways for repair of topoisomerase-induced damage will illuminate how cells can initially recognize an enzyme that requires the action of DNA repair pathways. In this work, we have assessed how the MRN complex interacts with other pathways that affect the protein component of a TOP2cc.

Our work strongly supports previous studies that showed that the MRN complex, specifically the endonuclease activity of MRE11, is a pathway for removing TOP2ccs from DNA. (Neale et al., 2005; Lee et al., 2012; Deshpande et al., 2016; Hoa et al., 2016). In addition, we show that this pathway functions for the removal of both isoforms, TOP2α and TOP2β. The endonuclease activity of MRE11 is insufficient and CtIP is also required. This is clearly seen in the results shown in Figure 7, where ectopic expression of MRE11 can lead to disjoining of TOP2ccs, even when proteasome activity is inhibited, but the disjoining fails in the absence of CtIP.

Previous work from our laboratory explored the importance of the MRE11 endonuclease in genome instability induced by TOP2ccs. We described a yeast Top2 mutant with elevated DNA cleavage. This mutation could not be viably expressed in yeast cells defective in homologous recombination such as *rad52* and *MreE11* null mutants (Stantial et al., 2020). Expression of the hypercleavage mutant in wild-type cells gave rise to short duplications that are dependent on non-homologous end-joining (NHEJ). Surprisingly, the hypercleavage mutant could be expressed in yeast cells carrying a mutation that eliminates Mre11p endonuclease activity. This result from yeast suggests that the ability to disjoin Top2ccs from DNA may not be the most important role of Mre11 in repairing Top2 damage. Interestingly, the levels of error-prone repair induced by the hypercleavage mutants in Mre11 nuclease-deficient strains are elevated. This observation suggests that at least in yeast, removal of TOP2cc by Mre11 leads to an error-free pathway of repair.

The importance of proteolysis of TOP2 prior to removal by MRE11 has not been carefully examined previously. The assay system of Keeney and colleagues (Neale et al., 2005) relied on intact immunoprecipitable TOP2 or TOP2-like proteins. Since they recovered intact Spo11 bound to DNA, their results suggested that proteolysis was not likely to be required for Spo11 processing, In the ICE assay shown in Figure 6, detection requires the epitope recognized by the antibody. Isoform-specific antibodies for TOP2 are directed against the (non-conserved) C -terminal domain and loss of the C terminus will prevent detection of trapped TOP2. Therefore, the lack of synergy in the experiment shown in Figure 6 may be affected by removal of the epitope recognized by the isoform-specific antibody. At present, isoform-specific antibodies with epitopes near the active site tyrosine are not available. Nonetheless, a simple interpretation of our result shown in Figure 6 is that normal levels of expression of MRE11 are insufficient to process Top2ccs. While the overexpression experiment shown in Figure 7 suggests that high levels of MRE11 can overcome the lack of proteolysis, our experiments do not take into account other non-proteasomal proteases such as SPRTN, nor other “disentangling” proteins such as ZATT (Schellenberg et al., 2017).

**FIGURE 10:**
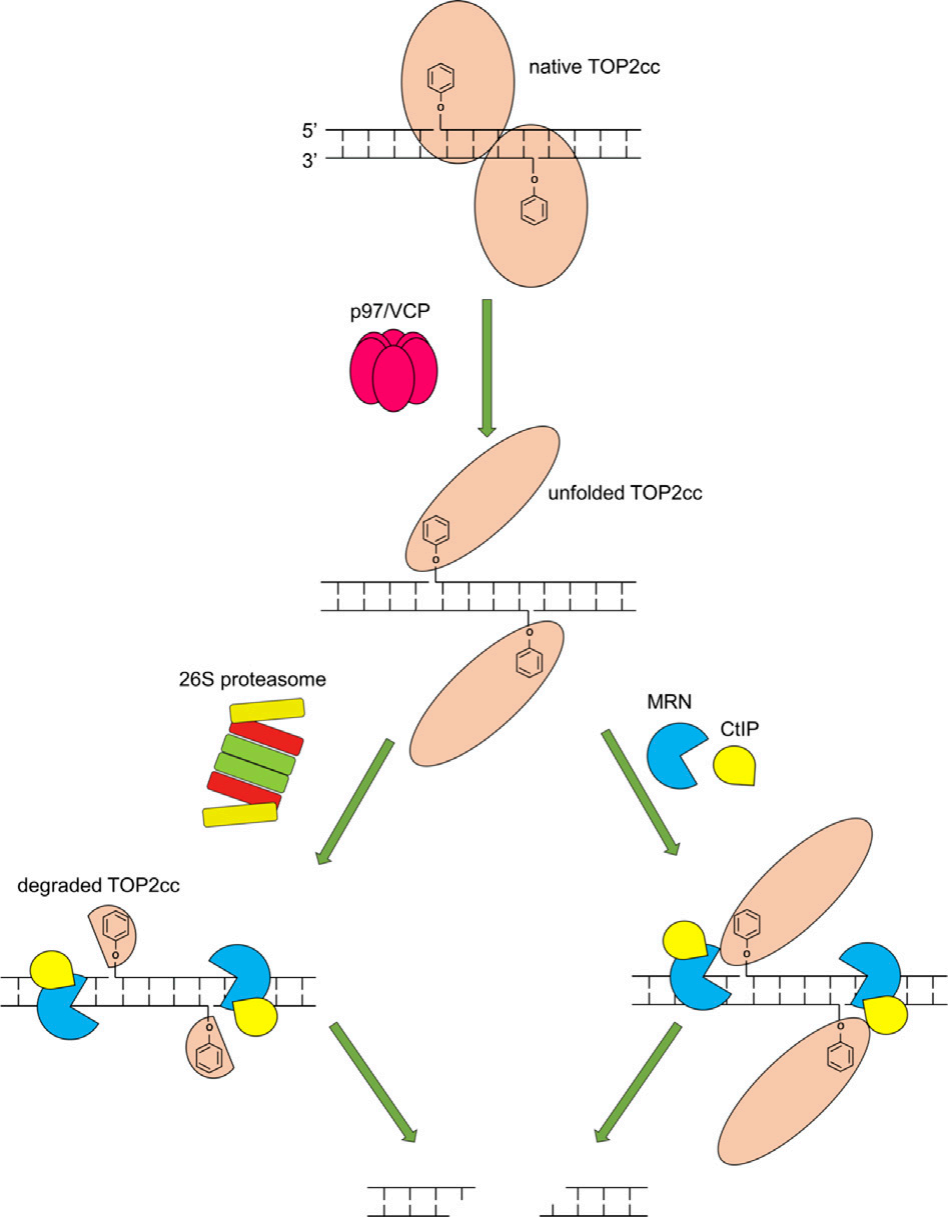
Model of requirements for the MRN complex to repair TOP2cc. Upon trapping of a TOP2cc, VCP/p97 is recruited and unfolds the TOP2cc, enabling the 26S proteasome (left) and the MRN complex and CtIP (right) to process the TOP2cc. The MRN complex in cooperation with CtIP incises the vicinity of the TOP2cc using its single-strand endonuclease activity, releasing the TOP2cc and liberating the otherwise TOP2-linked DSB (the release may require Mre11 3′-5′ exonuclease activity following its endonucleolytic incision). Proteolytic degradation of TOP2cc by the 26S proteasome may facilitate the nucleolytic processing by the MRN complex and CtIP.

A key finding in our work is that while MRE11 overexpression can overcome proteasome inhibition, it cannot overcome inhibition of the ATPase/segregase VCP/p97. Recent work has demonstrated that VCP/p97 is important for repairing TOP2-induced damage (Swan et al., 2021), and our results agree with those findings. Interestingly, we found that inhibition of VCP/p97 by NMS873 led to lower levels of TOP2ccs than inhibition of the proteasome using the inhibitor MG132 (Supplementary Figure S3), but that inhibition of VCP/p97 was epistatic to inhibition of the proteasome by MG132. These results taken together, along with our observation that VCP/p97 is required for MRE11 removal of TOP2ccs, indicate a complex pathway upstream of nucleolytic processing of TOP2ccs (Figure 10). We hypothesize that VCP/p97 cooperates with multiple proteases, both the proteasome, and other repair proteases such as Spartan (Reinking et al., 2020), GCNA (Dokshin et al., 2020) and the yeast protease DDI1 (Serbyn et al., 2020).

Our work highlights a biochemical approach to studying the repair of topoisomerase-induced DNA damage. The guiding hypothesis is that detection of elevated levels of TOP2ccs indicates a defect in disjoining trapped complexes. Other approaches for detecting elevated levels of TOP2ccs and based on similar biophysical principles have been described, including the TARDIS assay (Willmore et al., 1998) and the RADAR assay (Kiianitsa and Maizels, 2013). A detailed description of some of these assays along with detailed methodological considerations has recently been presented (Nitiss et al., 2021). The ICE assay has been previously used to demonstrate the importance of SUMO, ubiquitin and poly (ADP-ribose) modification of topoisomerases in repairing the enzyme-induced damage (Sun et al., 2020a; Sun et al., 2021; Sun et al., 2022b), potential roles of p53 in the regulation of repair functions for topoisomerase-induced damage (Menendez et al., 2022), and the identification of novel repair activities (JLN, unpublished data).

MRE11, RAD50, and NBS1 expressions as clinical biomarkers for cancer prognosis and responses to chemotherapies have been reported. High MRE11 expression has been implicated with poor prognosis and chemoresistance in gastric cancer, colon cancer, breast cancer as well as glioma (Altan et al., 2016; Bian et al., 2019; Maksoud, 2022). As MRE11 and NBS1 deficiencies sensitize human cancer cells to etoposide (Hoa et al., 2016), it is worth investigating combination with etoposide and the MRE11 endonuclease inhibitors (PFM 01 and 03) in cancer cell lines as well as tumor xenograft models with different MRE11 expressions. For example, chemo- and radio-resistant small cell lung cancer (SCLC) cells display a higher expression of the MRN complex (Li et al., 2021), targeting MRE11 using PFM 01/03 could potentially improve the response of these cells to etoposide, a key component of the first-line therapy for SCLC treatment.

Future work will require a more precise ordering of pathways mediated by VCP/p97 and the proteasome. Our work describes new assay systems that can be used for *in vitro* processing assays. Finally, we still lack detailed understanding of how a TOP2 protein becomes a substrate for repair. The recognition of a TOP2 trapped by a small molecule inhibitor, a TOP2 mutation, or a DNA structure alteration must have some properties that precipitate repair reactions, and the tools described in this paper will be particularly useful in understanding the unique properties of trapped TOP2ccs.

## Materials and methods

### Cell culture

RH30 and HEK293 cells were cultured in RPMI-1640 and DMEM media (Life Technologies), respectively, supplemented with 10% (v/v) fetal bovine serum, 100 U/ml penicillin, 100 μg/ml streptomycin and 1 × Glutamax in T-75 tissue culture flasks at 37°C in a humidified incubator with regulated CO_2_ at 5%). The RH30 cell line, a gift from Dr. Peter Houghton, University of Texas San Antonio, was verified by ATCC.

### siRNA and plasmid transfection

For siRNA knockdown studies, RH30 cells were transiently transfected with validated human MRE11 siRNA (Dharmacon), human NBS1 siRNA (Dharmacon), or negative control siRNA (Dharmacon D-001810-02 ON-TARGETplus Non-targeting siRNA) for 72 h using DharmaFECT transfection reagent (Dharmacon) following the manufacturer’s instructions. pICE-HA-MRE11 plasmid was a gift from Patrick Calsou (Addgene plasmid # 82033). The plasmid was transfected to cells using Lipofectamine 3000 (Thermo Fisher) for 48 h before drug treatments and ICE assays.

### MRE11 inhibitors treatments

Inhibition of MRE11 was accomplished by pre-treatment with various MRE11 inhibitors based on Mirin (Dupre et al., 2008). Inhibitors tested included PFM01 (an MRE11 endonuclease inhibitor), PFM03 (an MRE11 endonuclease inhibitor), or PFM 39 (an MRE11 exonuclease inhibitor). Inhibitors were synthesized as previously described (Shibata et al., 2014). Treatment with each of the inhibitors was for 4 h at 37°C, at a concentration of 25 μM. Assessment of effects of etoposide was performed by adding 10 μM etoposide for 2 h at 37°C in the continued presence of MRE11 inhibitors.

### Immunodetection of TOP2-DNA covalent complexes (ICE bioassay)

TOP2-DNA covalent complexes were isolated and detected using *in vivo* complex of enzyme (ICE) bioassay as previously described (Anand et al., 2018). Briefly, cells were lysed in 1% sarkosyl solution after drug or solvent exposure. Cell lysates were sheared through a 25 g needle (10 strokes) to reduce the viscosity of DNA; followed by layering atop a CsCl solution (1.5 g CsCl/ml). Followed by centrifugation in an NVT 90 rotor (Beckman coulter) at 42,000 RPM for 20 h at 25°C. The resulting pellet containing DNA, RNA and TOP2-DNA covalent complexes was obtained and dissolved in 1x TE buffer. After overnight incubation in TE buffer, the DNA concentration of the samples was measured using a UV spectrophotometer measuring absorbance at 260 nm (BioTek synergy 2 multi-mode reader). 2 µg samples were diluted with 25 mM NaPO_4_ (pH6.5) buffer and then applied to a nitrocellulose membrane using a slot-blot vacuum manifold (Bio-Rad). Typically, 2 μg of DNA was applied per sample. TOP2-DNA adducts were immunodetected using rabbit anti-TOP2α polyclonal antibody (1:1000, Bethyl, A300-054 A) and mouse anti-TOP2β monoclonal antibody (1:10000, BD Transduction Lab, 611492), followed by horseradish peroxidase (HRP)-conjugated secondary antibody (Cytiva NA931, Mouse IgG and Cytiva NA934, Rabbit IgG, Millipore Sigma) incubation and ECL detection. The level of TOP2-DNA covalent complexes was quantified by densitometric analysis of TOP2 cc signal using ImageJ software.

### Western blotting

Cell lysates were obtained for validation of siRNA knockdown. Proteins were quantified using Bio-Rad protein assay (Bio-Rad). Proteins (30 μg per lane) were separated through a 4–15% (w/v) precast SDS polyacrylamide gel (Bio-Rad) and transferred onto PVDF membrane (Bio-Rad). Blots were immunostained with anti-MRE11 (rabbit, 1:5000, Cell Signaling), anti-NBS1 (rabbit, 1:5000, Cell Signaling), followed by HRP-conjugated secondary antibodies and ECL detection. Anti-β-actin monoclonal antibody (mouse, 1:10000, Santa Cruz Biotechnology) was used as protein loading control.

### *In vitro* TOP2cc repair assays using ICE samples and purified MRN complex

HEK293 cells were treated with DMSO or 200 µM etoposide for 30 min, followed by ICE assay. 10 µg DNA sample isolated by ICE assay is incubated with recombinant 100 nM MRN and 100 nM CtIP in 1 × cleavage buffer (40 mM Tris–HCl pH 8.0, 60 mM NaCl, 5 mM MgCl2, 1 mM MnCl2, 2 mM DTT, 0.2 mg/ml BSA) at 37°C for 30 min. Samples were then mixed with laemmli buffer for Western blotting and the blots were immunostained with anti-TOP2α and mouse anti-TOP2β antibodies, followed by HRP-conjugated secondary antibodies and ECL detection.

### Statistical analysis

Error bars on bar graphs represent standard deviation (SD) and *p*-value was calculated using 2-tailed paired student’s t-test for independent samples. All ICE sample quantitation was performed with a minimum of three independent biological replicates.

### Reproducibility considerations

The standard protocol for quantitative ICE assays is to apply duplicate samples to nitrocellulose membranes. These duplicate samples are typically from separate CsCl gradients run at the same time. While each experimental condition was analyzed with three separate treatments performed on different days, most of the experimental conditions were examined more than three times. Experiments that showed significant differences between the technical duplicate samples were typically discarded. Experimental conditions including aspects such as etoposide concentrations were tested in pilot experiments, and not included among the three biological replicates.

## Data Availability

The original contributions presented in the study are included in the article/Supplementary Material, further inquiries can be directed to the corresponding author.

## Supplementary material

SUPPLEMENTARY FIGURE S1 Knockdown of CtIP by siRNA increases the level of etoposide-induced TOP2-DNA complexes as detected by ICE. The experimental conditions were the same as described in Figure 1 except that RH30 cells were treated with siRNA against CtIP. **(A)** Western blot assessing CtIP knockdown efficiency. **(B)** ICE assay illustrating TOP2αcc in CtIP knockdown cells. The panel illustrates a representative assay. **(C)** Densitometric analysis of all experiments comparing relative integrated densities of TOP2αcc signal amongst CtIP knockdown and control cells treated with 2 μM, 10 μM and 50 μM etoposide. Integrated density of TOP2αcc signal of each group was normalized to that of control cells treated with etoposide. **(D)** ICE assay illustrating TOP2βcc. **(E)** Densitometric analysis of all experiments comparing relative integrated densities of TOP2βcc signal amongst CtIP knockdown and control cells treated with 2 μM, 10 μM and 50 μM etoposide. Integrated density of TOP2αcc signal of each group was normalized to that of control cells treated with 2 μM etoposide.

SUPPLEMENTARY FIGURE S2 MRE11 exonuclease activity is not required for TOP2cc removal. RH30 cells were pre-treated with 25 μM PFM 03 or 25 μM PFM 39 (exonuclease inhibitor) for 4 hours. Etoposide (10 μM) was added, and incubation was continued for 2 hours. Cells were then harvested for ICE assays. **(A)** Representative ICE assay results detecting TOP2αcc. **(B)** Quantitation for TOP2αccs. Integrated density of TOP2αcc signal of each group was normalized to that of cells treated with etoposide alone. **(C)** Representative ICE assay results detecting TOP2βcc. **(D)** Quantitation for TOP2βccs.

SUPPLEMENTARY FIGURE S3 VCP/p97 inhibitor increases Top2ccs. NMS873 treatment is epistatic to proteasome inhibition. HEK293 cells were pre-treated with 10 μM NMS873 (VCP/p97 inhibitor), 10 μM MG132, or 10 μM NMS873 plus 10 μM MG132 for 1 hour. Etoposide (10 μM) was added, and incubation was continued for 2 hours. Cells were then harvested for ICE assays. **(A)** Representative ICE assay results detecting TOP2αcc. **(B)** Quantitation for TOP2αccs. Integrated density of TOP2αcc signal of each group was normalized to that of cells treated with etoposide alone. **(C)** Representative ICE assay results detecting TOP2βcc. **(D)** Quantitation for TOP2βccs.
