# Supplementary figures and images for: Requirements for MRN endonuclease processing of topoisomerase II-mediated DNA damage in mammalian cells
Source: Front Mol Biosci. 2022 Sep 23;9:1007064. doi: 10.3389/fmolb.2022.1007064 (PMC9537633; doi:10.3389/fmolb.2022.1007064)

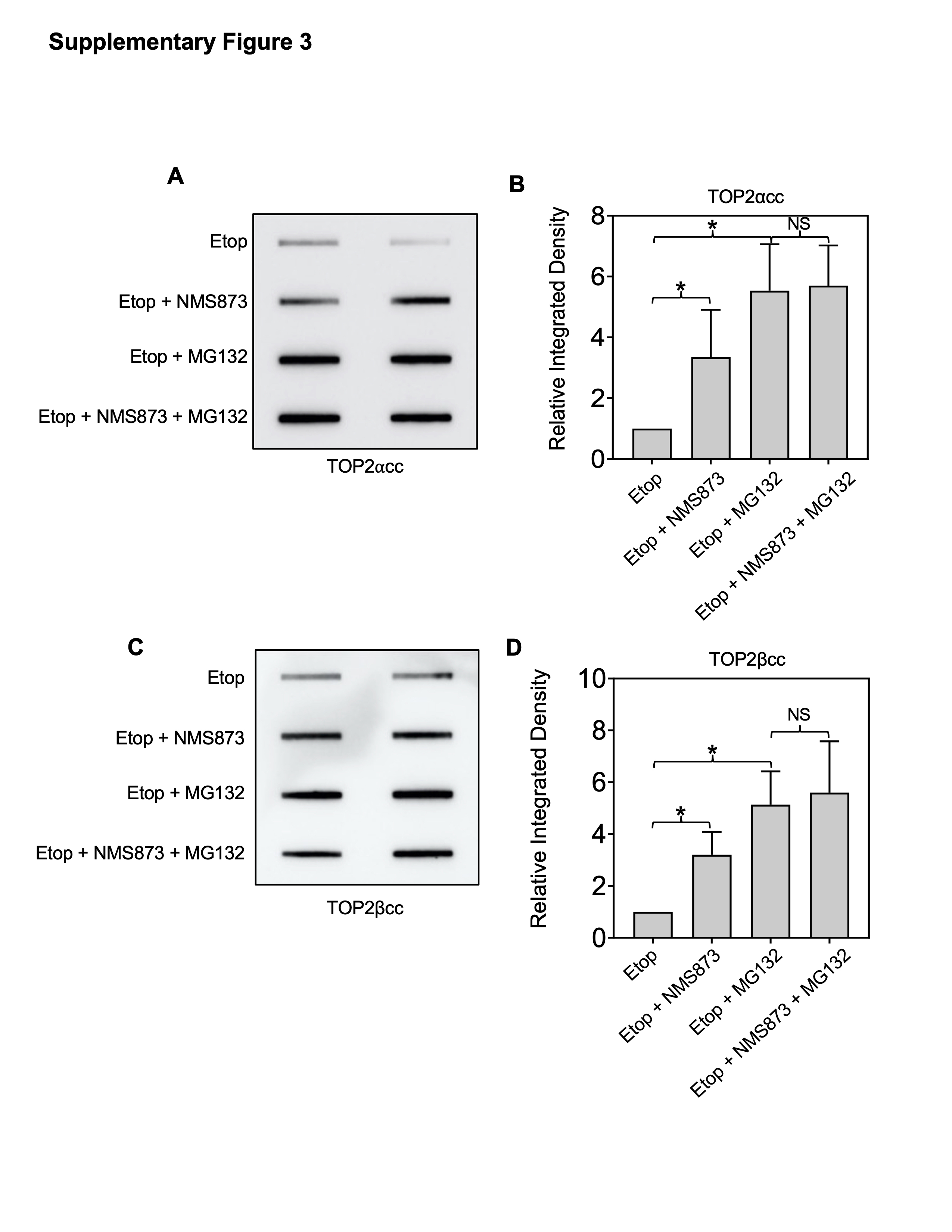

Supplement: Supplementary file 1 [file Image3.jpg]

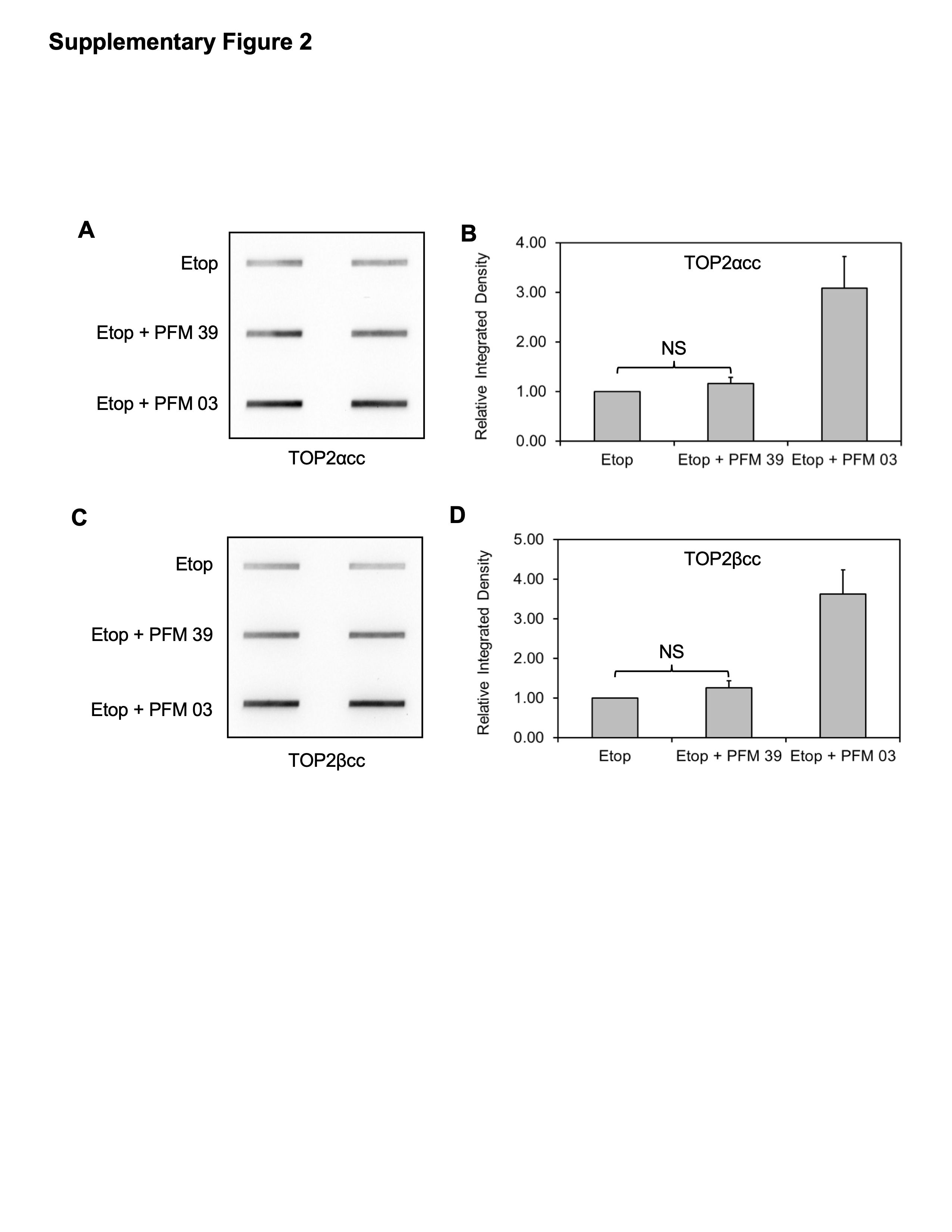

Supplement: Supplementary file 2 [file Image2.jpg]

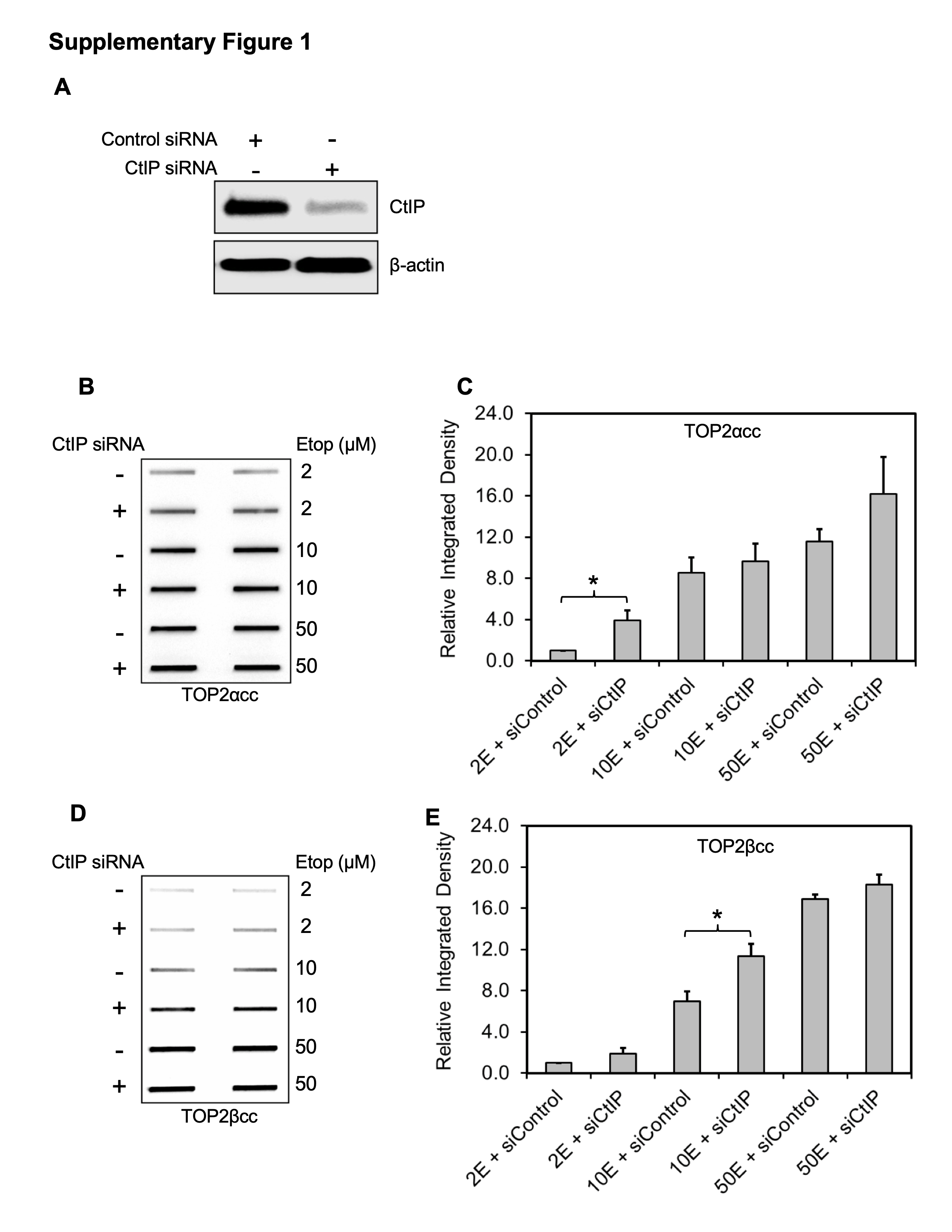

Supplement: Supplementary file 3 [file Image1.jpg]
